# The improvement of mechanical properties of conventional concretes using carbon nanoparticles using molecular dynamics simulation

**DOI:** 10.1038/s41598-021-99616-y

**Published:** 2021-10-12

**Authors:** Liang Zhao, Mahyuddin K. M. Nasution, Maboud Hekmatifar, Roozbeh Sabetvand, Pavel Kamenskov, Davood Toghraie, As’ad Alizadeh, Teimour Ghahari Iran

**Affiliations:** 1 School of Civil and Architectural Engineering, Yangtze Normal University, Chongqing, 408100 China; 2 DS & CI Research Group, Universitas Sumatera Utara, Medan, Indonesia; 3 Department of Mechanical Engineering, Khomeinishahr Branch, Islamic Azad University, Khomeinishahr, Iran; 4 Department of Energy Engineering and Physics, Faculty of Condensed Matter Physics, Amirkabir University of Technology, Tehran, Iran; 5 Department of Propaedeutics of Dental Diseases, Sechenov First Moscow State Medical University, Moscow, Russia; 6 Department of Mechanical Engineering, Urmia University, Urmia, Iran; 7 Department of Mechanical Engineering, College of Engineering, University of Zakho, Zakho, Iraq

**Keywords:** Engineering, Mathematics and computing

## Abstract

In the present study, the improvement of mechanical properties of conventional concretes using carbon nanoparticles is investigated. More precisely, carbon nanotubes are added to a pristine concrete matrix, and the mechanical properties of the resulting structure are investigated using the molecular dynamics (MD) method. Some parameters such as the mechanical behavior of the concrete matrix structure, the validation of the computational method, and the mechanical behavior of the concrete matrix structure with carbon nanotube are also examined. Also, physical quantities such as a stress–strain diagram, Poisson's coefficient, Young's modulus, and final strength are calculated and reported for atomic samples under external tension. From a numerical point of view, the quantities of Young's modulus and final strength are converged to 35 GPa and 35.38 MPa after the completion of computer simulations. This indicates the appropriate effect of carbon nanotubes in improving the mechanical behavior of concrete and the efficiency of molecular dynamics method in expressing the mechanical behavior of atomic structures such as concrete, carbon nanotubes and composite structures derived from raw materials is expressed that can be considered in industrial and construction cases.

## Introduction

Concrete is a broad concept that refers to any material or compound that is composed of a cementations adhesive. Concrete can be made from different types of cement as well as sulfur, additives, polymers, fibers, etc. Also, heat, water vapor, vacuum, hydraulic pressures, and various compressors may be used in its construction^1^. Sometimes to change some of the concrete properties, when mixing materials, the amount of additives is added to it. These additives can also be in nanoscale. The nature of nanotechnology means working at the molecular and atomic levels in the dimensions of 1–100 nm^2–4^. Nanotechnology is used to intervene and fabricate the arrangement of devices, materials, and particles for greater efficiency^5^. Carbon nanotubes are one of the most important and widely used carbon structures. In addition to being very strong, carbon nanotubes also have good flexibility. From an atomic point of view, carbon nanotubes are cylindrical molecules with open or closed ends. In general, carbon nanotubes can be divided into three general categories: zig-zag carbon nanotubes, chiral carbon nanotubes and armchair carbon nanotubes^6,7^. Armchair carbon nanotubes share electrical properties similar to metals. Existence armchair carbon nanotubes in concrete increase the flexural and tensile compressive strength. In the construction industry, the presence of carbon nanotubes in concrete increases the strength and prevents the penetration of cracks^8^. In a computational study, Wu et al.^9^ simulated the mechanical behavior of a concrete sample. In this research, the molecular dynamics method and LAMMPS software were used for this purpose so that the field of selected forces in this work was selected as Compass, UFF, and Dreiding, and the results of each potential were compared with experimental results. The results of molecular dynamics simulations showed the high accuracy of all three force fields in predicting the mechanical properties of concrete structures. Yu et al.^10^ investigated the mechanical properties of concrete using the coarse-grained method. In this study, concrete particles were considered as disk-type particles, and the Gay-Bourne force field was used to predict their mechanical properties. The results of the research were in good agreement with the experimental results. Tavakoli et al.^11^ studied the mechanical properties of different phases of concrete and determined its properties by examining concrete with atomic precision and simulating it with nanometer dimensions. In this study, eight different force fields were used to perform simulation MD instruments, and mechanical factors such as Young's modulus, shear modulus, compressibility, and final strength of atomic structures were calculated and reported. Titscher et al.^12^ designed high-density concrete using molecular dynamics simulation. In this computational work, they studied the mechanical properties of condensed concrete structure and showed that this secondary structure has promising mechanical properties. Zhou et al.^13^ simulated concrete with a polymer chain. They reported the samples' mechanical properties by examining the interaction between concrete and simulated polymer chains so that the mechanical and thermodynamic results of this group showed an improvement in mechanical behavior and increased strength of the simulated structure. Hassan et al.^14^ added single-walled carbon nanotubes to conventional concrete samples. By examining the mechanical factors such as the strength of the nanocomposite, they reported the amount of improvement in the mechanical performance of this structure compared to the concrete matrix. Manzur et al.^15^ added multilayer carbon nanotubes into the concrete matrix. By adding multi-walled carbon nanotubes into the concrete matrix, the improvement of the mechanical properties of the obtained material was proved. Carriço et al.^16^ added multi-walled carbon nanotubes to the concrete sample. By adding carbon nanotubes with a weight ratio of 0.05–0.1%, they estimated the chemical stability and mechanical strength of these structures at 21–25%. By adding carbon nanotubes with mass ratios of 0.2%, 0.4%, and 0.6% to the concrete structure, Evangelista et al.^17^ investigated the change in mechanical properties of these structures. The results of the mechanical experiments show that the maximum improvement in the mechanical properties of the nanocomposition created for 0.4% of carbon nanotubes added. With the help of carbon nanotubes and carbon nanofibers, Danoglidis et al.^18^ proposed a new method to improve the mechanical properties of concrete samples. In this experimental study, they increased the amount of modulus of nanocomposites by adding carbon nanostructures. In addition to the molecular dynamics method, there are other methods for studying micro- and nano-scale currents^19–21^.

**Figure 1:**
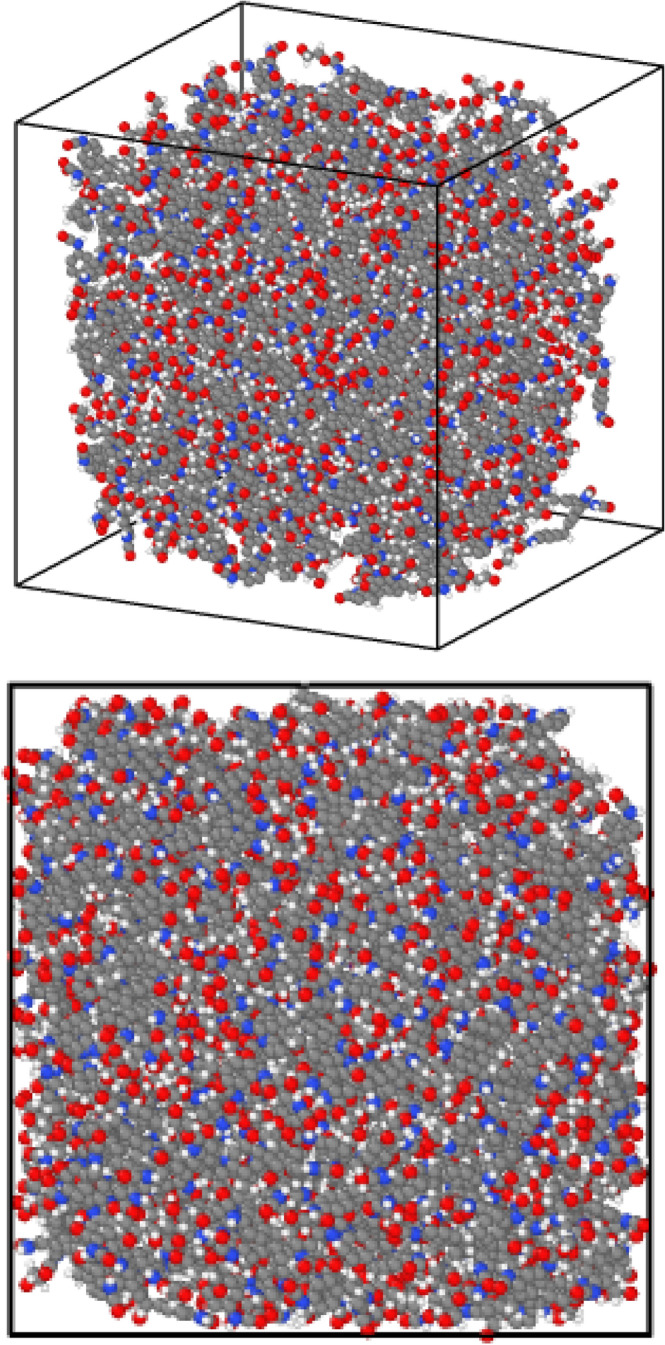
View of the atomic structure of a simulated concrete matrix.

In this paper, the computer simulations and molecular dynamics method are used. To apply the molecular dynamics method in the present study, LAMMPS software is used ^22^. The MD simulations tend to have high strain rates and the mechanical parameters are strain rate dependent^23^. In this research, parameters such as the temperature of the simulated system, the total energy of the simulated system, the mechanical behavior of the concrete matrix structure, the validation of the computational method, and the concrete matrix structure's mechanical behavior carbon nanotube were studied. Initially, in this study, a matrix of concrete containing the atoms of calcium, hydrogen, oxygen, and silicon is created inside the simulation box. In the next step, carbon nanotubes with different types (armchair, zig-zag and chiral) are simulated. In the third step, the composition of the concrete matrix with carbon nanotubes is determined with certain ratios and then the final atomic system is equilibrated at initial temperature as initial condition. The simulations of this research are performed in two general steps of equilibration of atomic structures and mechanical testing of pure concrete structure and reinforced concrete with carbon nanotubes. The first part of the simulated structures is done in 1,000,000 time steps and in the second stage the simulation process continues until the complete stress–strain diagram of the structure is obtained. To be more precise, in the calculations which performed in this research, the results reported in each of the simulation steps are sampled once every 10,000 time steps, so that the each time step is considered equal to 1 fs (Δt = 1 fs). An example of a simulated primary concrete structure with present atoms in the concrete structure is shown in Fig. 1.

## Computational method

In molecular dynamics simulation, each atom's acceleration in the system can be determined by having the force applied to each atom. Then, by integrating the equation of motion, the path of motion of the particles, which describes the location, speed, and acceleration of each particle, is determined over time. Thus the evolution of the path of motion over time will be determined. In the next step, using the path of movement, the average values ​​of the properties can be determined. In the molecular dynamics method, all calculations are performed according to Newton's equations, and in the simulation, Newton's second law formulation is used as below equation^24–27^.

1 $$F_{i} = \sum\limits_{i \ne j} {F_{ij} = m_{i} \frac{{d^{2} r_{i} }}{{dt^{2} }} = m_{i} \frac{{dv_{i} }}{dt}}$$

In this equation, F_i_ is the total force in simulated structures, r_i_/v_i_ is position/velocity of atoms, and m_i_ represents the atomic mass. Computationally, The velocity-Verlet algorithm is used to integrate Newton's law equation^28^.

In MD simulations, force-fields have high importance to describe physical behavior of atomic systems. Here, the Lennard–Jones potential function is used to describe the interactions between concrete particles as well as the interactions between carbon nanotube particles and concrete. The most common relationship for the Lennard–Jones potential function is as follows^29^:

2 $${U}_{LJ}=4\varepsilon \left[{\left(\frac{\sigma }{r}\right)}^{12}-{\left(\frac{\sigma }{r}\right)}^{6}\right] r<{r}_{c}$$

In this relation, *ε* is the depth of the well, *σ* is the distance at which the potential value becomes zero, and *r* is the distance of the particles from each other. Also, r_c_ is cut-off radius in Eq. (2). This parameter value set to 12 Å in our MD study. Based on the particles present in the simulation box, the used coefficients in this research are shown in Table 1.

**Table 1:** Lennard–Jones potential coefficients are used in this study.

Atom type	ε (kcal/mol)	(Å)σ
Si	0.402	4.295
Ca	0.238	3.399
O	0.06	3.500

In general, for carbon structures, the manner of interaction between atoms is described using the Tersoff potential function and with the following formulation^30^:

3 $$E \, = \, \raise.5ex\hbox{$\scriptstyle 1$}\kern-.1em/ \kern-.15em\lower.25ex\hbox{$\scriptstyle 2$} \sum\limits_{i} {\sum\limits_{i \ne j} {V_{i} } }$$

4 $$U_{ij} = f_{c} (r_{ij} )[f_{R} (r_{ij} ) + b_{ij} f_{A} (r_{ij} )]$$

In this respect, $f_{A}$ is a numerically negative value. The expression $f_{R}$ is also numerically positive and indicates the repulsion interaction in simulated carbon systems. Electrical potential energy, or electrostatic potential energy, is the potential energy obtained from the *Coulomb* steady forces. Electric potential energy is defined using *Coulomb* law as follows^31^:

5 $$U_{ij} \left( r \right) = \frac{ - 1}{{4\pi \varepsilon_{0} }}\frac{{q_{i} q_{j} }}{{r_{ij}^{2} }}$$

In the above relation, q_i_ and q_j_ are electric charges, *r* is the distance between the charges, and $\varepsilon_{0}$ indicates the electrical permeability of the open space. After force-field description, mechanical behavior details are important. In the molecular dynamics method and LAMMPS software, achieving and calculating the strain created in the structure is obtained by defining a variable and equating it with the strain formulation as follows:

6 $$Strain = \frac{{l_{2} - l_{1} }}{{l_{1} }}$$

where l_1_ and l_2_ are initial and final length of simulated structures in mechanical deformation process. Also, the amount of stress is also calculated with Eq. (7):

7 $$Stress = \frac{F}{A}$$

In this equation, F is atomic force and A defines cross section of simulated structure. Computationally, in the simulations performed here, the MD box length is 100 × 100 × 100 Å^3^ in x, y, and z-directions, respectively. Periodic boundary conditions are applied in all three directions of the coordinate axes so that this matrix is repeated in all three directions to infinity. Also, NVE and NVT ensembles are used to initial condition setting in atomic structures. Technically, in the present study, the Nose–Hoover thermostat is used to create thermal equilibrium in simulated atomic structures. In general, the thermostat is used to keep the temperature in a particular range. This process takes place by controlling the flow of thermal energy into or out of the simulated atomic structure^32,33^. Based on the descriptions given in this section, MD simulations in our computational research consist of 2 main steps:

*Step A) Equilibration process of atomic structures*: Firstly, the pristine/reinforced concrete matrix was simulated for 1,000,000 time steps. In this step, Nose–Hoover thermostat was used to equilibrate of atomic system temperature at T_0_ = 300 K as initial condition. Temperature damping ratio in this step is equal 0.01. For equilibrium process detecting, physical parameters such as temperature and total energy were reported.

*Step B) Mechanical behavior of atomic structures:* Next, the mechanical deforming and pullout process was implemented to pristine/reinforced matrix by using the NVE ensemble for 10,000 time steps. After the tensile and pullout tests, physical parameters such as stress–strain curve, Young’s modulus, ultimate strength, and interaction energy were reported to describe the mechanical behavior of pristine/reinforced concrete matrixes.

## Results and discussion

### Investigation of thermodynamic equilibrium in the simulated atomic structure

**Figure 2:**
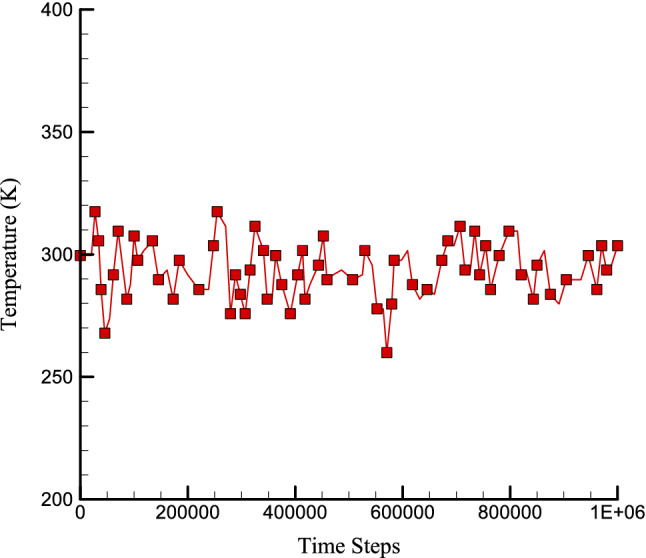
Temperature changes of pristine concrete matrix versus time.

**Figure 3:**
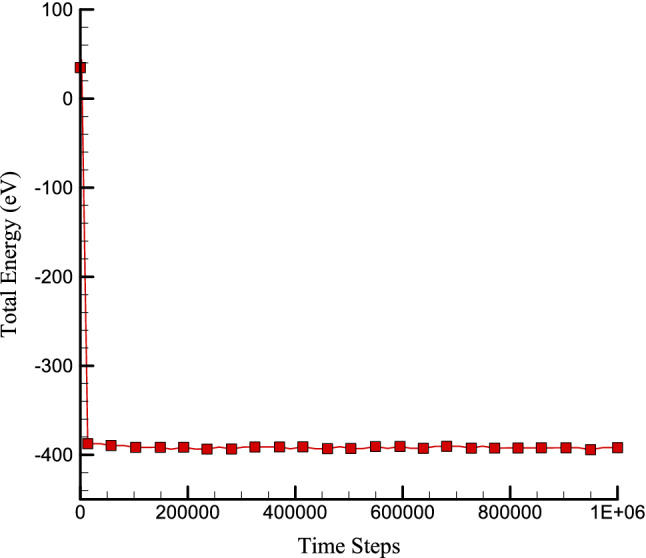
Total energy changes versus time for pristine concrete matrix.

**Figure 4:**
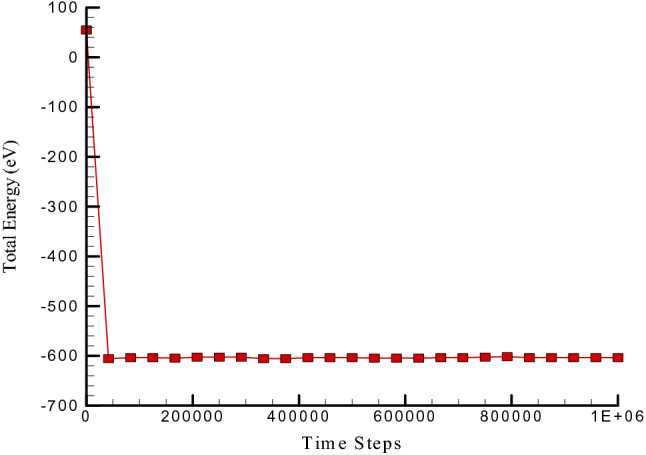
Total energy changes versus time for reinforced concrete matrix with zig-zag nanotube.

In the first step of this research, the structure of concrete is simulated, and the thermodynamic equilibrium in this structure is investigated. To investigating the thermodynamic equilibrium in this atomic structure, physical properties such as the temperature of the simulated system and the energy of the whole simulated system are studied. According to the calculation method, the diagrams corresponding to the temperature and energy of the whole structure are shown in Figs. 2 and 3. Based on the graph of temperature changes drawn in Fig. 2, the temperature in atomic structures tends to 300 K, indicating the temperature equilibrium in the simulated atomic structures. Figure 3 also shows the changes in total energy over time steps. The total energy in the simulated structures is equal to the sum of the atomic structures' kinetic energy and potential. Due to the convergence of these two quantities, it is expected that the total energy in these structures will also converge. The convergence of this quantity in a negative value indicates that the atomic structure's potential energy is greater than its kinetic energy. Total energy of concrete matrix in presence of zig-zag nanotube calculated to report the CNT effects on atomic behavior of pristine structure. As depicted in Fig. 4, by CNT inserting to pristine matrix, total energy magnitude of atomic structure increases and converge to -603.77 eV after 1000000 time steps. This atomic stability increasing in MD box arises from attraction force enlarging between various atoms. So, we conclude, CNTs adding to concrete matrix don’t disrupted initial atomic arrangement.

### Investigation of mechanical behavior of concrete matrix structure

**Figure 5:**
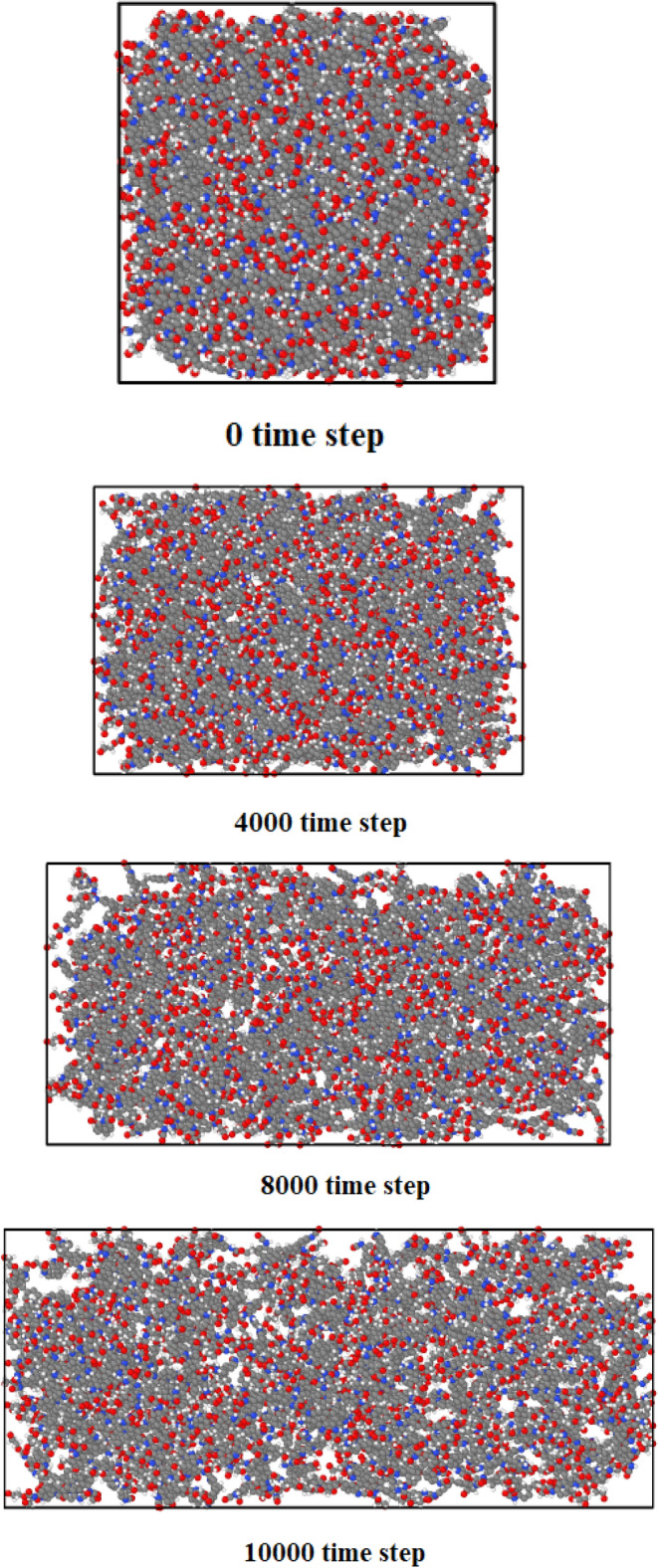
Mechanical traction process of concrete in the initial time step.

In this part of the research, and after ensuring the correctness of the simulations performed in the field of modeling atomic structures using LAMMPS software, we calculate the mechanical properties of the simulated concrete sample using the molecular dynamics method. The basis for calculating the mechanical properties in the simulation is the use of the loading method and deforms order in LAMMPS software, in which the stress-strain diagram is calculated, and mechanical information is extracted by creating tension in the simulated structure. In this method, the structure's initial length is calculated according to the number of atoms present in the simulation. Then, by stretching the structure in the next moments using the structure's instantaneous length in the strain formula, the strain value is calculated. Also, the stress-strain diagram of the simulated structure is calculated. The mechanical traction process created in the simulated atomic structures is shown in Fig. 5. In this mechanical procedure, the length of atomic matrix changes in z-direction. Numerically, simulated structure deformed by 0.01 s^−1^ strain rate with Δt=1 fs and with NVE ensemble used in z direction. This atomic deformation process implemented to all regions of atomic sample and all atoms of modeled matrix deformed in each computational steps.

### Computational method validation

**Figure 6:**
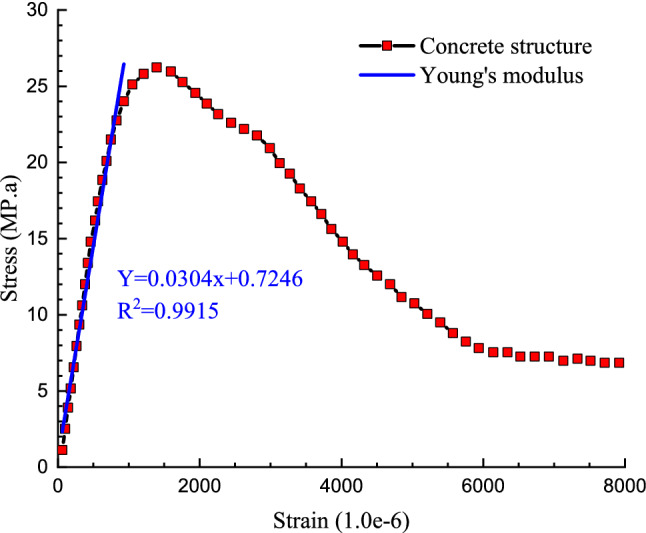
Calculated stress–strain and Young’s modulus diagrams for concrete structure using molecular dynamics method.

In this part of the research, by performing molecular dynamics simulations and tensile tests in the simulated atomic structure, the initial concrete structure's stress and strain values are obtained. By drawing the corresponding values ​​obtained for the concrete structure's strain and stress, the stress-strain diagram of the simulated structure is obtained. This diagram is shown in Fig. 6. The reported results for stress values in this figure averaged every 1000 time steps to reduce fluctuation in this calculated parameter. According to the diagram that is drawn in this figure, the maximum stress in the simulated atomic structure is equal to 26.21 MPa, which is in good agreement with experimental research results^34^. From a numerical point of view, the maximum stress tolerance by different concrete samples is in the range of 20–40 MPa. All calculations and reports made in this section are done in standard conditions. This indicates the efficiency of the molecular dynamics method in simulating the mechanical properties of atomic structures such as a concrete matrix. From a physical and technical point of view, the appropriate results obtained in this study are simulated due to the appropriate selection of interatomic interactions in structures and also the allocation of suitable atomic positions for different atoms in the concrete structure, which shows the exact method of atomic modeling. From a mechanical point of view, the linear region in the stress-strain diagram contains important information such as Young's modulus of the atomic structure is simulated so that by calculating the slope of the stress-strain diagram drawn in the linear region, it is possible to calculate Young's modulus by simulation. The numerical value of Young's modulus calculated for concrete was 30 GPa (Fig. 6). On the other hand, by creating a deformation in the structure of the concrete sample in the *x* and *y* directions, it is possible to calculate Poisson's coefficient in the atomic structure of concrete. This quantity's numerical value is calculated to be 0.15, which is also consistent with previous research^34^. To be more precise, the experimental reports in this field express the numerical value of Poisson's coefficient in standard conditions for concrete in the range of 0.1–0.2, which is in good agreement with the results obtained in the simulations dissertation^34^.

### Investigation of mechanical behavior of concrete matrix structure with carbon nanotubes

**Figure 7:**
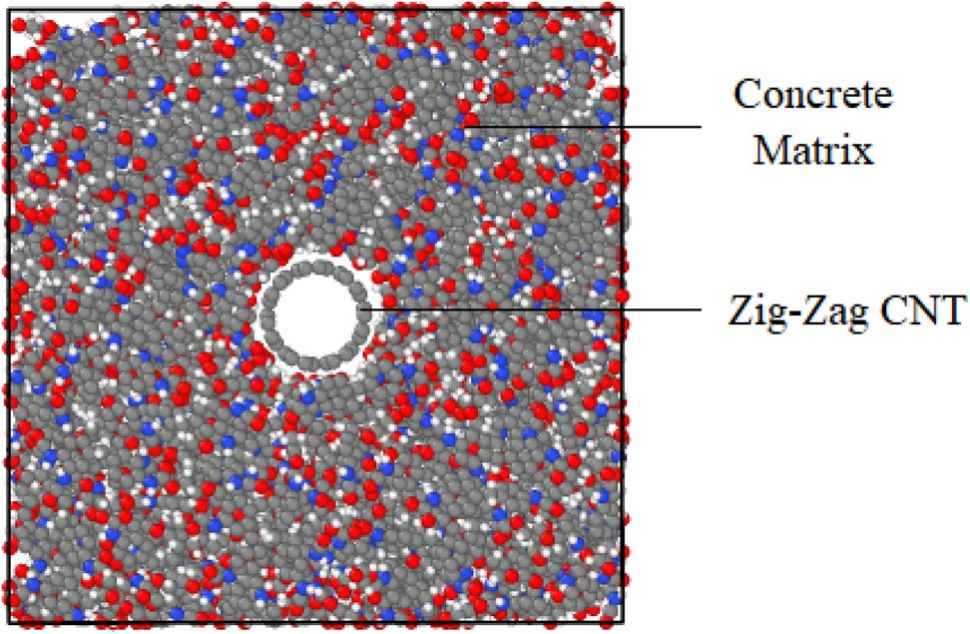
A view of the atomic structure of a concrete matrix with simulated carbon nanotubes in the present study.

**Figure 8:**
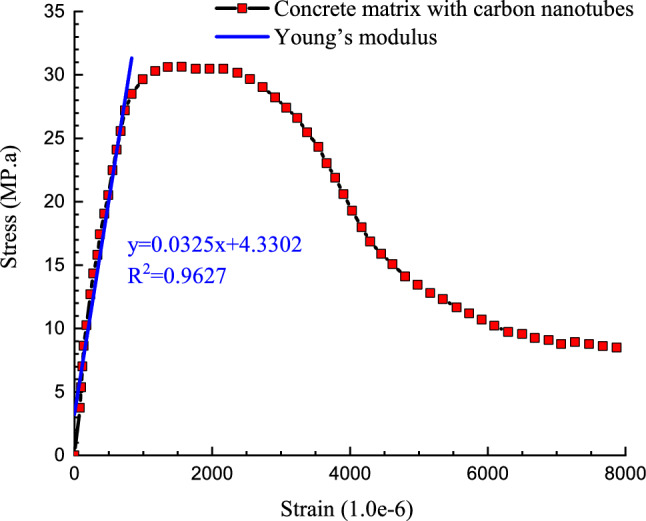
Calculated stress–strains and Young’s modulus diagram for the structure of concrete matrix with zig-zag carbon nanotubes.

By adding carbon nanotubes with the armchair, zig-zag, and chiral edges into the concrete matrix, the strength of the initial atomic sample increases. This factor can be described by examining the stress-strain diagrams of atomic structures in Figs.7 and 8. As reported before, the results for stress values in stress-strain curve averaged every 1000 time steps to reduce computational fluctuation. Based on the obtained results in this part of the research, the addition of carbon nanotubes with zig-zag edges has the greatest impact on the mechanical behavior of the concrete sample. This behavior arises from mechanical strength of CNTs with various edges. Between CNT structures, nanotubes with zig-zag edge have higher mechanical strength and this factor increase mechanical behavior of reinforced matrix, appreciably. From an atomic point of view, carbon atoms are present in the structure of carbon nanotubes with the zig-zag edge has larger adsorption interaction than chiral and armchair samples. This adsorption interaction cause more physical stability in nanostructure against external parameters.

**Figure 9:**
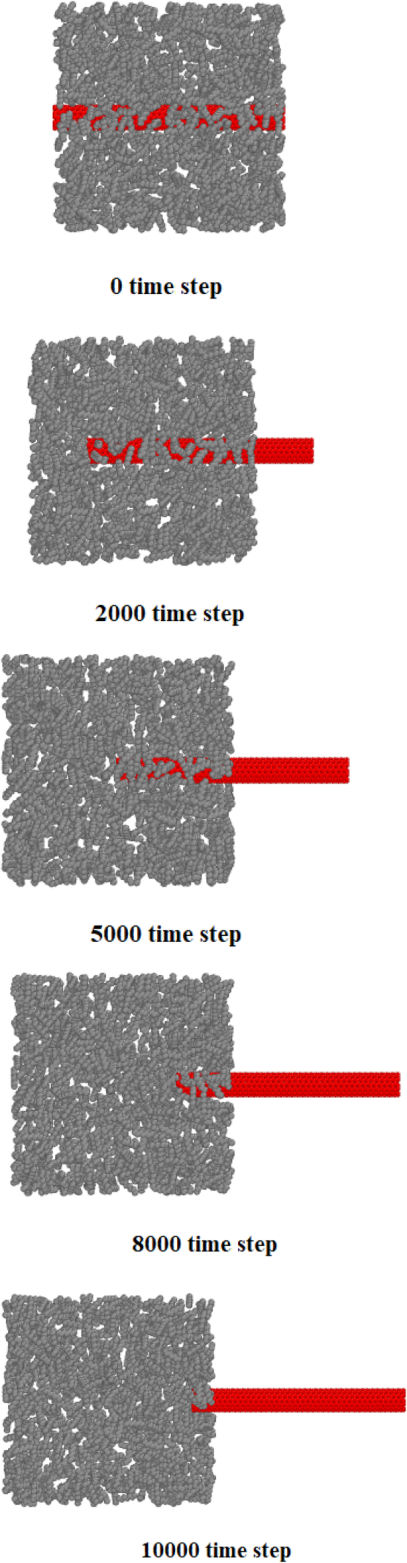
Schematic diagram of the CNT pullout process from the pristine matrix as a function of MD simulation time steps.

**Figure 10:**
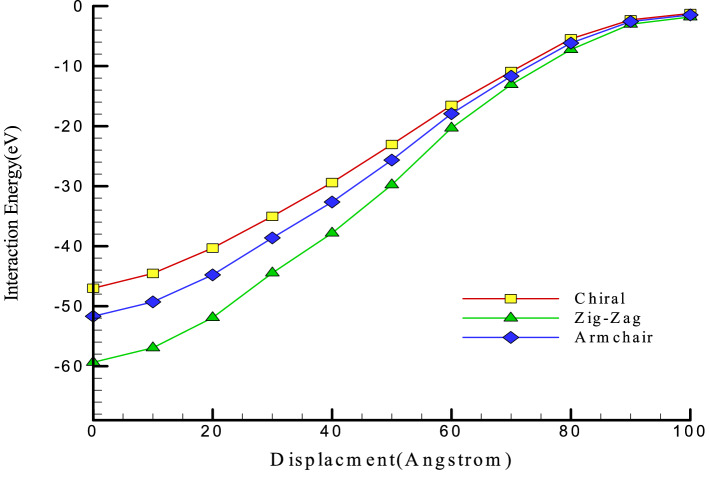
Interaction energy between various CNTs and a pristine matrix during the atomic pullout process.

For attraction force analyze in simulated structures, we calculated the interaction energy between atoms in various CNT structures and pristine matrix. This process was implemented on the equilibrated compound of reinforced concrete/CNT structure containing CNTs with armchair, zigzag, and chiral edges (separately). In these MD simulations, the periodic condition from the xy plane change to fix one and fixing the concrete structures at the back region of the CNT for 10 Å depth. The nanotube was pull out along the z axis for 10000 time steps, while the interaction energy between two atomic structures computed throughout the pullout procedure as depicted in Fig. 9. The calculated energy as a function of atomic displacement is reported in Fig. 10. Numerically, interaction energy for carbon atoms in armchair, zig-zag, and chiral nanotubes is − 51.68 eV, − 59.36 eV, and − 47.01 eV. So, this calculation shows more atomic stability in CNT structure with zig-zag edge. Technically, interaction energy in simulated structures was calculated by using “compute group/group” command in LAMMPS computational package. As a result, the mechanical strength of this structure is higher than in these samples. From a numerical point of view, according to the numbers reported in Table 2, it indicates an increase in the amount of Young's modulus and ultimate strength to numerical values ​​of 32 GPa and 30.34 MPa. From a numerical point of view, the mechanical quantities reported in this section have increased by 6.67% and 15.76%, respectively. As a result, the use of carbon nanotubes can be one of the most suitable solutions to improve the mechanical properties of concrete. It is worth mentioning that the results in this part of the research are obtained for the addition of carbon nanotubes with two atomic percentages so that the chirality of the carbon nanotubes used in this part is selected in such a way that the atomic percentage expressed in this part is equal to 2% in all samples. By using this atomic percentage, the probability of nanoparticles collision with each other decreases and the behavior of the pristine matrix improve appreciably. In the final step of current computational study, we report the potential energy of simulated structures in presence of various CNTs. MD results in this step show the potential energy of pristine matrix increases from − 423.87 eV to − 551.01 eV, − 571.97 eV, and − 603.77 eV by chiral, armchair, and zig-zag nanotubes adding to concrete matrix. This calculation shows the physical stability improvement of atomic structures which cause mechanical behavior improvement in final compound. By done these final calculations, we conclude simulated structures in current computational work can be used in actual applications^35–39^.

**Table 2:** Calculated mechanical properties for concrete matrix specimens using molecular dynamics method.

Atomic sample	Ultimate strength (MPa)	Young's modulus (GPa)	Poisson's ratio	Interaction energy (eV)
Pristine concrete matrix	26.21	30	0.15	–
Concrete with zig-zag nanotubes	30.34	32	0.17	− 59.36
Concrete with armchair nanotubes	29.29	31	0.16	− 51.68
Concrete with chiral nanotubes	29.01	31	0.16	− 47.01

## Conclusion

In this study, using computer simulations, the mechanical properties of the improved concrete matrix were investigated using the addition of carbon nanotubes. The simulations performed in this study were of the molecular dynamics type, which was performed using LAMMPS software. The results of this research can be expressed as follows:

- The amount of temperature in atomic structures tends to 300 K, which indicates the temperature equilibrium in the simulated atomic structures.
- The convergence of total energy in a negative value indicates that the potential energy of the atomic structure is greater than its kinetic energy, and as a result, the higher potential energy than the kinetic energy of the concrete structure indicates its physical equilibrium and mechanical stability.
- Young’s modulus was determined by simulation using a stress–strain diagram. The numerical value of the calculated Young's modulus for concrete was 30 GPa.
- By creating a deformation in the structure of the concrete sample in the *x* and *y*- directions, it is possible to calculate Poisson's coefficient in the atomic structure of concrete. The numerical value of this quantity was calculated to be 0.15.
- The addition of carbon nanotubes with a zig-zag edge has the greatest effect on the mechanical behavior of concrete samples because carbon atoms in the structure of carbon nanotubes with a zig-zag edge have a stronger adsorption interaction than chiral and armchair samples.

## References

[1] Madina B, Gumilyov LN (2020). Determination of the most effective location of environmental hardenings in concrete cooling tower under far-source seismic using linear spectral dynamic analysis results. J. Res. Sci. Engi. Technol..

[2] Mosavi A, Hekmatifar M, Alizadeh AA, Toghraie D, Sabetvand R, Karimipour A (2020). The molecular dynamics simulation of thermal manner of Ar/Cu nanofluid flow: the effects of spherical barriers size. J. Mol. Liquids.

[3] Toghraie, D., Hekmatifar, M., & Jolfaei, N. A. Investigation of heat transfer and fluid flow behaviors of CuO/(60: 40)% ethylene glycol and water nanofluid through a serpentine milichannel heat exchanger. *Int. J. Numer. Methods Heat Fluid Flow* (2019).

[4] Abu-Hamdeh, N. H., Almatrafi, E., Hekmatifar, M., Toghraie, D., & Golmohammadzadeh, A., Molecular dynamics simulation of the thermal properties of the Cu-water nanofluid on a roughed Platinum surface: Simulation of phase transition in nanofluids. *J. Mol. Liquids* 114832 (2020)

[5] Toghraie D, Hekmatifar M, Salehipour Y, Afrand M (2019). Molecular dynamics simulation of Couette and Poiseuille Water-Copper nanofluid flows in rough and smooth nanochannels with different roughness configurations. Chem. Phys..

[6] Muhammad Adnan A (2018). A theoretical study of the size effect of carbon nanotubes on the removal of water chemical contaminants. J. Res. Sci. Engi. Technol..

[7] Aghili A, Kamrani MR (2021). Modeling of the thermal degradation of poly (methyl methacrylate) and its nanocomposite with multi-walled carbon nanotubes. Adv. Appl NanoBio-Technologies.

[8] Bethune D, Kiang CH, De Vries M, Gorman G, Savoy R, Vazquez J, Beyers R (1993). Cobalt-catalysed growth of carbon nanotubes with single-atomic-layer walls. Nature.

[9] Wu W, Al-Ostaz A, Cheng AH-D, Song CR (2011). Computation of elastic properties of Portland cement using molecular dynamics. J. Nanomech. Micromech..

[10] Yu Z, Lau D (2015). Nano-and mesoscale modeling of cement matrix. Nanoscale Res. Lett..

[11] Tavakoli D, Tarighat A (2016). Molecular dynamics study on the mechanical properties of Portland cement clinker phases. Comput. Mater. Sci..

[12] Titscher T, Unger JF (2015). Application of molecular dynamics simulations for the generation of dense concrete mesoscale geometries. Comput. Struct..

[13] Zhou S, Vu-Bac N, Arash B, Zhu H, Zhuang X (2019). Interface characterization between polyethylene/silica in engineered cementitious composites by molecular dynamics simulation. Molecules.

[14] Hassan A, Elkady H, Shaaban IG (2019). Effect of adding carbon nanotubes on corrosion rates and steel-concrete bond. Sci. Rep..

[15] Manzur, T., Yazdani, N., Emon, M., & Bashar, A., Potential of carbon nanotube reinforced cement composites as concrete repair material. *J. Nanomater. *2016 (2016).

[16] Carriço A, Bogas J, Hawreen A, Guedes M (2018). Durability of multi-walled carbon nanotube reinforced concrete. Constr. Build. Mater..

[17] Evangelista ACJ, de Morais JF, Tam V, Soomro M, Di Gregorio LT, Haddad AN (2019). Evaluation of carbon nanotube incorporation in cementitious composite materials. Materials.

[18] Danoglidis, P., & Konsta-Gdoutos, M., Reinforcing concrete with carbon nanotubes and carbon nanofibers: a novel method to improve the modulus of elasticity. pp. 98–99.

[19] Zajmi, L., Ahmed, F.Y., & Jaharadak, A.A. Concepts, methods, and performances of particle swarm optimization, backpropagation, and neural networks. *Appl. Comput. Intell. Soft Comput.*. 10.1155/2018/9547212 (2018).

[20] Tjahjono, T., Elveny, M., Chupradit, S., Bokov, D., Hoi, H. T., & Pandey, M. Role of cryogenic cycling rejuvenation on flow behavior of ZrCuAlNiAg metallic glass at relaxation temperature. *Trans. Indian Inst. Met.*10.1007/s12666-021-02395-3 (2021).

[21] Ozkaya, S.G., Baygin, M., Ozdemir, M.A., & Kazaz, I. Image processing based analysis of the compressive strength for the stones used in historical masonry structures. *Int. J. Comput. Sci. Software Eng.*.**6**(10), 216–222 (2017).

[22] Brown WM, Wang P, Plimpton SJ, Tharrington AN (2011). Implementing molecular dynamics on hybrid high performance computers–short range forces. Comput. Phys. Commun..

[23] Wang X, Huang X, Gao M, Zhao Y-P (2021). Mechanical response of kerogen at high strain rates. Int. J. Impact Eng..

[24] Schlick, T., Pursuing Laplace’s vision on modern computers. Math. Approach. Biomol. Struct. Dyn. 219–247 (1996)

[25] Bernal JD (1964). The Bakerian lecture, 1962. The structure of liquids. Proc. R. Soc. Lond. Ser. A Math. Phys. Sci..

[26] Alder BJ, Wainwright TE (1959). Studies in molecular dynamics. I. General method. J. Chem. Phys..

[27] Gibson J, Goland AN, Milgram M, Vineyard G (1960). Dynamics of radiation damage. Phys. Rev..

[28] Verlet L (1967). Computer" experiments" on classical fluids. I. Thermodynamical properties of Lennard-Jones molecules. Phys. Rev..

[29] Lennard-Jones JE (1931). Cohesion. Proc. Phys. Soc..

[30] Tersoff J (1988). New empirical approach for the structure and energy of covalent systems. Phys. Rev. B.

[31] Huray PG (2011). Maxwell's equations.

[32] Nosé S (1984). A unified formulation of the constant temperature molecular dynamics methods. J. Chem. Phys..

[33] Hoover WG (1985). Canonical dynamics: Equilibrium phase-space distributions. Phys. Rev. A.

[34] Ma Q, Guo R, Zhao Z, Lin Z, He K (2015). Mechanical properties of concrete at high temperature—A review. Constr. Build. Mater..

[35] Alirezaie A, Hajmohammad MH, Hassani Ahangar MR, Hemmat Esfe M (2018). Price-performance evaluation of thermal conductivity enhancement of nanofluids with different particle sizes. Appl. Therm. Eng..

[36] Oveissi, S., Eftekhari, S.A. & Toghraie, D. Longitudinal vibration and instabilities of carbon nanotubes conveying fluid considering size effects of nanoflow and nanostructure. *Physica E.* **83**, 164–173 (2016).

[37] Hemmat Esfe M, Bahiraei M, Mahian O (2018). Experimental study for developing an accurate model to predict viscosity of CuO–ethylene glycol nanofluid using genetic algorithm based neural network. Powder Technol..

[38] Hemmat Esfe M, Karimpour R, Abbasian Arani AA, Shahram J (2017). Experimental investigation on non-Newtonian behavior of Al_2_O_3_ -MWCNT/5W50 hybrid nano-lubricant affected by alterations of temperature, concentration and shear rate for engine applications. Int. Commun. Heat Mass Transfer.

[39] Hemmat Esfe M, Abbasian Arani AA, Firouzi M (2017). Empirical study and model development of thermal conductivity improvement and assessment of cost and sensitivity of EG-water based SWCNT-ZnO (30%:70%) hybrid nanofluid. J. Mol. Liq..

