# Editorial Expression of Concern: The improvement of mechanical properties of conventional concretes using carbon nanoparticles using molecular dynamics simulation

**DOI:** 10.1038/s41598-022-15653-1

**Published:** 2022-07-05

**Authors:** Liang Zhao, Mahyuddin K. M. Nasution, Maboud Hekmatifar, Roozbeh Sabetvand, Pavel Kamenskov, Davood Toghraie, As’ad Alizadeh, Teimour Ghahari Iran

**Affiliations:** 1 School of Civil and Architectural Engineering, Yangtze Normal University, Chongqing, 408100 China; 2 DS & CI Research Group, Universitas Sumatera Utara, Medan, Indonesia; 3 Department of Mechanical Engineering, Khomeinishahr Branch, Islamic Azad University, Khomeinishahr, Iran; 4 Department of Energy Engineering and Physics, Faculty of Condensed Matter Physics, Amirkabir University of Technology, Tehran, Iran; 5 Department of Propaedeutics of Dental Diseases, Sechenov First Moscow State Medical University, Moscow, Russia; 6 Department of Mechanical Engineering, Urmia University, Urmia, Iran; 7 Department of Mechanical Engineering, College of Engineering, University of Zakho, Zakho, Iraq

Editorial Expression of Concern to: *Scientific Reports* 10.1038/s41598-021-99616-y, published online 12 October 2021

The Editors are issuing an Editorial Expression of Concern to alert readers that this article shows substantial indication of irregularities in authorship during the submission process. Readers should also note that the source of the coefficients used in Table 1 was omitted and is Rappé et al. 1992^1^.

Davood Toghraie agrees with this statement. Liang Zhao, Mahyuddin K. M. Nasution, Maboud Hekmatifar, Roozbeh Sabetvand, Pavel Kamenskov, As’ad Alizadeh & Teimour Ghahari Iran have not responded to correspondence about this statement.
